# A neural coding scheme reproducing foraging trajectories

**DOI:** 10.1038/srep18009

**Published:** 2015-12-09

**Authors:** Esther D. Gutiérrez, Juan Luis Cabrera

**Affiliations:** 1 Laboratorio de Dinámica Estocástica, Centro de Física, Instituto Venezolano de Investigaciones Científicas. Caracas 1020-A, Venezuela

## Abstract

The movement of many animals may follow Lévy patterns. The underlying generating neuronal dynamics of such a behavior is unknown. In this paper we show that a novel discovery of multifractality in winnerless competition (WLC) systems reveals a potential encoding mechanism that is translatable into two dimensional superdiffusive Lévy movements. The validity of our approach is tested on a conductance based neuronal model showing WLC and through the extraction of Lévy flights inducing fractals from recordings of rat hippocampus during open field foraging. Further insights are gained analyzing mice motor cortex neurons and non motor cell signals. The proposed mechanism provides a plausible explanation for the neuro-dynamical fundamentals of spatial searching patterns observed in animals (including humans) and illustrates an until now unknown way to encode information in neuronal temporal series.

In the context of animal movement the earliest reference to the superdiffusion of organisms is a study by Shlesinger and Klafter^1^ about Lévy random walks (1986). Since this first theoretical prediction many studies have evidenced that the movement of several animals follow Lévy patterns. It is the case of movement patterns of wandering albatrosses^2^, jackals^3^, reindeers^4^, microzooplankton^5^, swarming bacteria^6^, spider monkeys^7^,^8^, root-feeding insects^9^,^10^, honey bees^11^, goats^12^, fresh water fishes^13^, snails^14^, fruit flies^15^, bony fish, sharks, sea turtles, penguins^16^,^17^,^18^, fallow deers^19^ and humans^20^, just to name a few. According to optimal foraging theory^21^,^22^, evolution through natural selection has led over time to highly efficient searching strategies. Remarkably, it has been shown that for the case of revisitable targets Lévy search patterns optimizes the searching process^23^. Indeed, many works aim at identifying the ecological scenarios under which Lévy patterns emerge (or not)^18^. The study of search patterns in organisms is an active research area important not only in the field of movement ecology but also because of political, economic, environmental, and health-related reasons^24^. As a matter of fact, biological searching is a particular instance of a random search. Random searches can include quite different situations like searches performed by enzymes on DNA strands for specific sequences^25^. An authoritative account of this problem can be found in^24^.

A reduced number of works have advanced ideas regarding internal biological dynamics producing these search patterns. Examples include anomalous diffusion as a result of stochastic time delayed dynamics in the nervous system^26^, adaptive memory losses of previous behavior^27^ or suppression of the scale-free power law behavior of fruit flies by blocking of synapses in the motor cortex^28^. In this context, one can ask if there are neuronal codification processes underlying the generation of animal search patterns. To the best of our knowledge this question is still unanswered, the underlying generating neuronal dynamics is currently unknown. In this work, we describe a plausible process for the neuro-dynamical origins of animal Lévy searches. The proposed mechanism is rooted in the so-called winnerless competition (WLC) dynamics, also known as cyclic dominance. We will show that WLC establishes a theoretical ground that facilitates information extraction from real neuronal dynamics that is translatable into Lévy search patterns.

Let us recall that WLC is grounded on heteroclinic cycles, i.e., on a collection of solution trajectories connecting equilibriums, periodic solutions, or chaotic invariant sets via saddle-sink connections. In WLC all participant agents alternate sequentially in time^29^,^30^ in such a way that the system outcome can be considered a coding sequence^30^. WLC has been suggested as an archetypal dynamical principle useful to explain a diverse range of nervous responses^31^,^32^,^33^. WLC remarks the importance of itinerant and competitive dynamics in the brain as an information processing device. Experimental and theoretical work provides evidence that transient states can better represent information processing in the brain^34^,^35^,^36^,^37^,^38^,^39^. An interesting example of the application of the WLC principle is the case of the mollusc *Clione limacina*, where it is used to understand the competitive dynamics between statocyst receptor neurons and how these deliver control signals for swimming and hunting^40^.

## Results

### Detecting multifractality in WLC residence times

**Figure 1:**
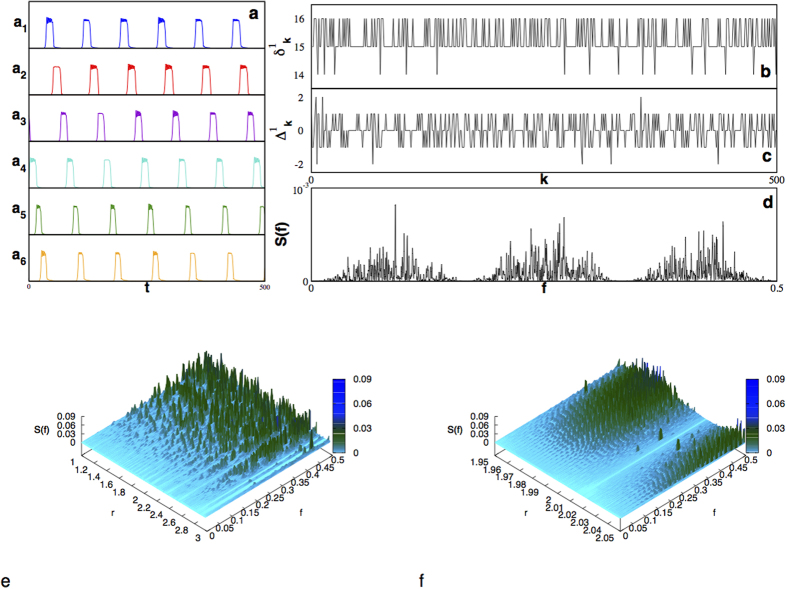
Scheme II processing of the temporal series and the power spectra for the difference between residence times on the (*r*, *f*)-plane. (**a**) Temporal series showing winnerless sequential alternation of activation between different neurons for Eq. 1. (**b**) Temporal behavior of the time spent in the vicinity of the saddle point , 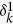. Note the complex variability of the saddle residence times. (**c**) Difference between residence times according to scheme II 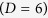. (**d**) Power spectrum for a temporal series as in (**c**). (**e**) Power spectra for the set of *r* values satisfying necessary and sufficient conditions for winnerless competition and a parameter step 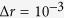. (**f**) Zoom on the subset of values 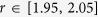 and a parameter step 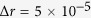.

**Figure 2:**
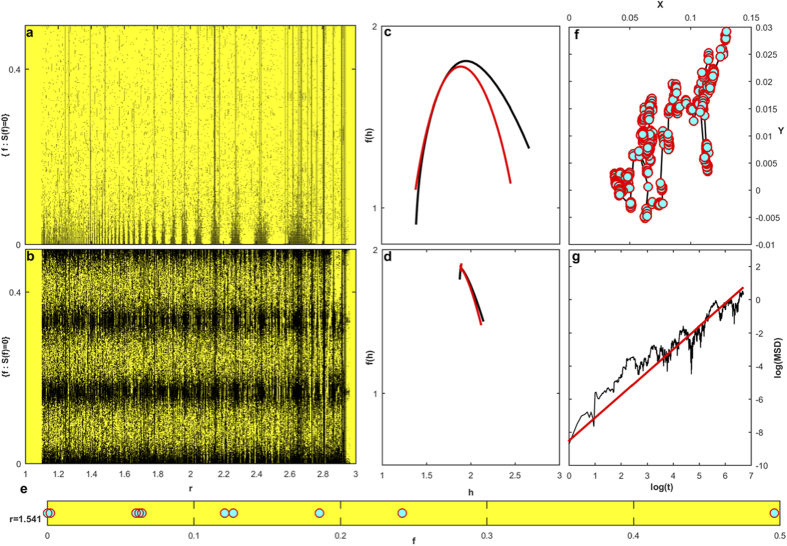
Hidden Multifractality. The set of points on the plane (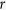, *f*) where the power spectrum reveals no frequency content (black points), describe multifractal patterns both for (**a**) scheme I and (**b**) scheme II. These results are for neuron *i* = 1 with 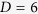, *r* was scanned with a step 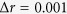. (**c**) Multifractal singularity spectra, 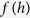 vs. *h*, is determined for scheme I (black line) and scheme II (red line), while (**d**) no multifractality is detected for the set of points with nonzero frequency content, independently of the scheme used. (**e**) From the multifractal pattern and a fix value of the control parameter, 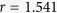, a fractal dust can be drawn. (**f**) Fractal dust gaps induce a superdifussive random walk on the plane with 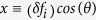 and 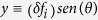; 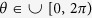 and 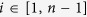. (**g**) It superdiffuse with a Hurst exponent 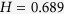 calculated from the mean squared displacement of (**f**) (black line) and the generalized Hurst exponent (red line).

To set up the theoretical background on which this work is based let us consider the set of *N* coupled Lotka-Volterra (L-V) maps given by the equation


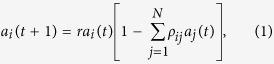


where 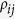 represents the strength of the inhibitory action of *i* on *j*, 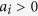 represents the activity of each neuron, 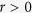 is a control parameter and 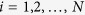 is the neuron index. Necessary and sufficient conditions for the existence of heteroclinic cycles supporting WLC for different values of *N* and *r* are known^41^. An illustrative temporal series showing WLC’s characteristic sequential alternation of the activation between different neurons can be observed in Fig. 1a. To extract novel encoding possibilities from WLC let us proceed as follows: i) Given a particular neuron *i* and the time series 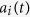, we calculate the time spent in the vicinity of the saddle point 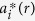, i.e., the residence time in the *k*-th visit, 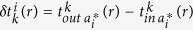, where 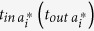 is the time spent approaching (departing) the neighborhood of 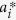. This time can be accessed simply measuring the width of an activation peak. With this information we can build the series of the times, 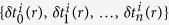, until an arbitrary visit index 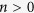. An illustrative example of this series is depicted in Fig. 1b. ii) At this point, the differences between residence times of visits to the saddle, separated by a time interval 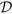, is calculated as 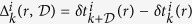. New temporal series can be assembled according to (at least) two different schemes: I) the temporal series calculating 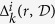 for an specific saddle and a fix value of the displacement parameter,


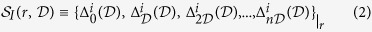


and II) the temporal series calculating 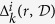 for an specific saddle point and the same value of the displacement parameter, but built including all the activation peaks,


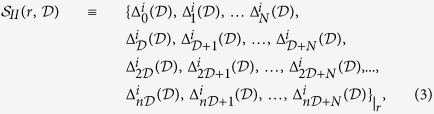


for arbitrary *n*. Note that these temporal series depend on the value of *r*. A particular result obtained applying scheme II is shown in Fig. 1c. The frequency content of the resulting oscillations is revealed by the power spectrum as is shown in Fig. 1d. This is a result obtained for a fixed value of the control parameter *r*. Therefore, we can calculate the power spectrum for all the values of the control parameter sustaining WLC behavior^41^, i.e., in the range 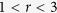. The result of this calculation is displayed in Fig. 1e . It can be observed that for certain values of *r* there seems to be no frequency content, i.e., the differences between residence times are constant. It has been demonstrated^41^ that residence times may show different regimens. In particular, they can display regimes of constant increments with the activation time (*k*), yielding constant differences between residence times. An additional situation producing constant differences corresponds to periodic residence times in which the constant equals to zero. Now, going back to Fig. 1e, it can be seen that Δ*r*-regions with nonzero frequency display bands containing Δ*r*-regions without frequency content. When observed in a smaller scale, a similar arrangement is found, evoking a certain ordering concealed on the 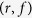 space (Fig. 1f). To analyze such a potential ordering we proceed by submitting the patterns on the 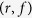 plane to a multifractal analysis. On the 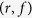-plane two different set of points were analyzed: i) 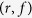 points where 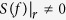 and ii) 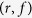 points where 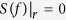. The 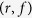-planes for schemes I and II are shown in Fig. 1a,b, respectively. In this representation, the black points correspond to values of *r* and *f* where 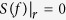. These sets of points were analyzed using the Chlabra *et al*. method for the calculation of the singularity spectrum^42^. Multifractality is determined plotting the Hausdorff dimension 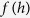 versus the Hölder exponent *h*^43^. A multifractal shows a characteristic 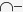 shape curve as those depicted in Fig. 2c. The resulting singularity spectra for sets i) and ii) and both schemes, reveals multifractality only for the set of points corresponding to 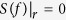 (see Fig. 2c,d), using both schemes. This outcome is robust when considering different values of *N* and under changes in the precision of *r*.

### Unveiling the neural code

We have been able to show hidden multifractality in WLC residence times. If transient sequential dynamics is subjacent to neuronal information processing^31^, it seems logical to us to enquire how this multifractality could encode neuronal information. We address this question in the following lines. Let us start at Fig. 2e, there we show the fractal dust, that we will denote by 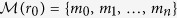, for a given 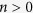. Such fractal dust has been extracted from the multifractal depicted at Fig. 2b, given an arbitrarily chosen fixed value of the control parameter, 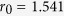. Each 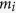 represents a frequency value where 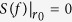. As the precision used to calculate the power spectrum is increased 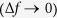 it is possible to detect more values of *f* satisfying the condition 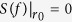. Thus, a fine-grained dust is approached asymptotically. The fractal dust shown in Fig. 2e has fractal dimension 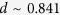. Now, lets consider the possibility of 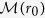 being a coding pattern, where the difference in the position of the dust points, the gap 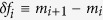 , 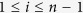, carries useful functional information. With this idea in mind we can built a random walk on the plane such that 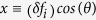, 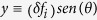, were *θ* (*i*) is arbitrarily selected from a random uniform distribution in the interval 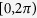 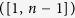. Figure 2f shows the resulting random walk. It is characterized by a short scale spatial exploration alternated with jumps covering larger scales. These movement yields a Hurst exponent 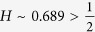 (Fig. 2g), i.e., it is a superdiffusive movement. We stress that not all sets 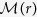 display the fractal structure required to yield a superdiffusive pattern. The reported pattern makes necessary tuning of the control parameter *r*.

**Figure 3:**
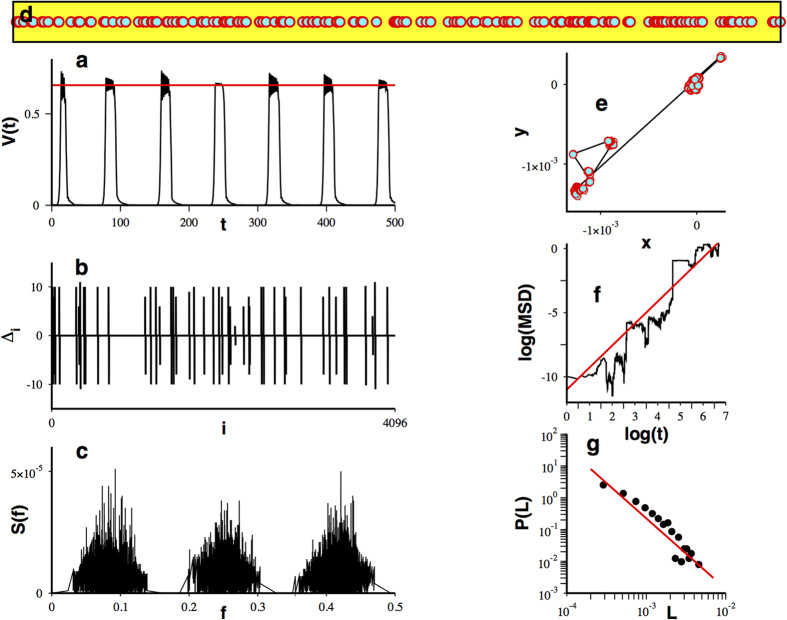
Lévy walks can be obtained from bursting temporal series. (**a**) Temporal series showing bursting behavior for Eq. (1) with 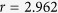. To measure spike’s width an arbitrary threshold is established (red line). (**b**) Difference between residence times on each constituent bursting spike (spike’s widths), according to scheme II 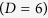. (**c**) Power spectrum of a temporal series calculated with 2^16^ points as shown in (**b**). (**d**) A subset of fractal dust for 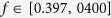 obtained from the power spectrum. (**e**) Induced random walk on the plane. (**f**) Mean squared displacement versus time calculated as in Figure 2(g) and (**g**) distribution of steps lengths for the induced random walk (**e**).

As we said before, WLC dynamics is rooted in the existence of heteroclinic cycles. These trajectories can connect not only equilibrium points but also more complex solutions, as periodic or chaotic sets, producing temporal bursting behavior. We would like to know if the approach used above can also account for cases where bursting behavior is present. In such a case, instead of dealing with the difference between the residence times we would be dealing with the difference between the residence times in each one of the cycles composing a complex oscillation, i.e., the width of each constituent bursting spike. To better explain this case consider the temporal series obtained with Eq. (1) for the particular case of 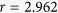 (Fig. 3a). This signal shows evident bursting behavior. By establishing an arbitrary edge value (red line in Fig. 3a) we can determine the width of each constituent bursting spike and with this information we can proceed to calculate the difference between those widths (Fig. 3b) according to scheme II. After calculating the power spectrum for this temporal series (Fig. 3c) we extract a fractal dust, as shown in Fig. 3d. The fractal dimension of this dust is 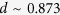. Indeed, this fractal induces a bidimensional random walk (Fig. 3e) with a temporal evolution of its mean squared displacement characterized by a Hurst exponent 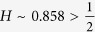 and a distribution of steps characterized by an exponent 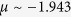 (Fig. 3g). This case exemplifies how a superdiffusive Lévy walk can be obtained from the underlying WLC multifractality. Note that the analysis of bursting spikes yields larger temporal series, therefore facilitating a proper estimation of the characteristic exponent.

**Figure 4:**
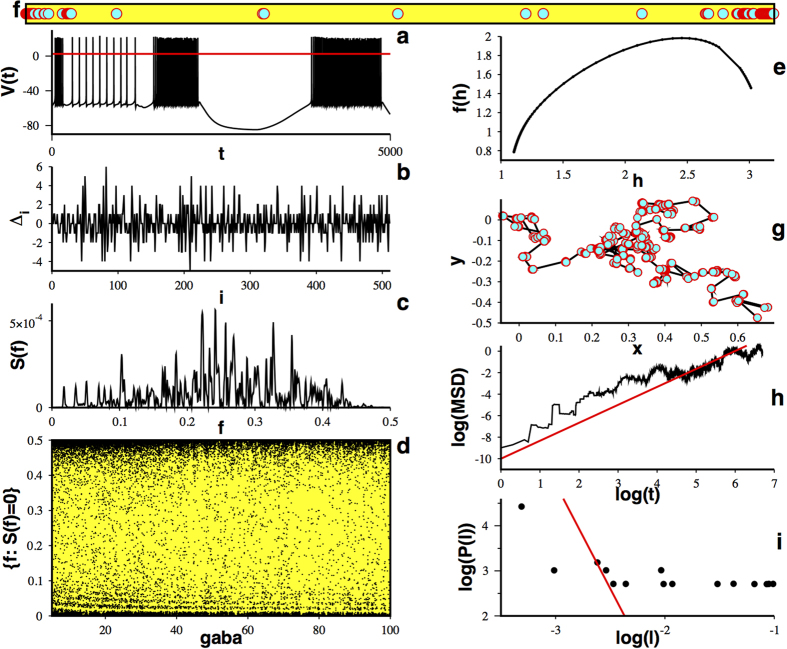
Multifractal induced superdiffusion in a conductance model. (**a**) Temporal series showing bursting of neural activity in the conductance model (used threshold in red). (**b**) Temporal behavior of the differences 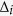 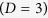. (**c**) Power spectra calculated with 2^10^ points for (**b**). (**d**) (Black) points satisfying 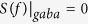 on the plane (*gaba*, *f*), (yellow) points not satisfying such a condition. (**e**) Multifractal singularity spectra for the set of black points. (**f**) A fractal dust determined from the cero frequency points of (**d**) for a fix value of *gaba*. (**g**) Induced superdiffusive random walk and its (**h**) mean squared displacement calculated as in Figure 2(g). (**i**) Steps distribution (best fit in red).

At this stage we have shown how a superdiffusive Lévy pattern can be obtained from WLC multifractality. However, our demonstration was not done using a realistic neuronal model. To overcome this limitation we consider a conductance based neuronal model also showing WLC dynamics^44^. Details about the model and its simulation can be found in the Supplementary Information. The conductance based model was analyzed with the same procedure as with the L-V map. Results for the scheme II are reported here. The temporal series obtained from the conductance model is shown in Fig. 4a and the series corresponding to the difference in residence times for visits separated a time interval 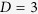, is depicted in Fig. 4b. The observed behavior is qualitatively similar to the one obtained from the differences in the case of the L-V map. The power spectrum for this signal is depicted in the Fig. 4c (calculated with 2^10^ data points). We calculated power spectra for different values of the model parameter *gaba* and, following the same treatment as with the L-V model, the set of values 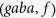, satisfying 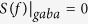, are represented on the plane, as depicted in Fig. 4d. A multifractal analysis reveals multifractality for the set of points satisfying 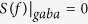 (see Fig. 4e). In particular, for a fix value of *gaba* = 50 nsec, we can extract a fractal dust with dimension 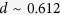, as shown in Fig. 4f. This fractal allows for the construction of a random walk (Fig. 4g) whose mean squared displacement obeys a power law with a Hurst exponent 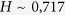. The distribution of steps does not show a promising shape. Even so, we fitted a power law to this collection of points obtaining an exponent 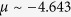. Clearly, this result is not conclusive of Lévy superdiffusion.

### Decoding Lévy walks from experimental neuronal data

**Figure 5:**
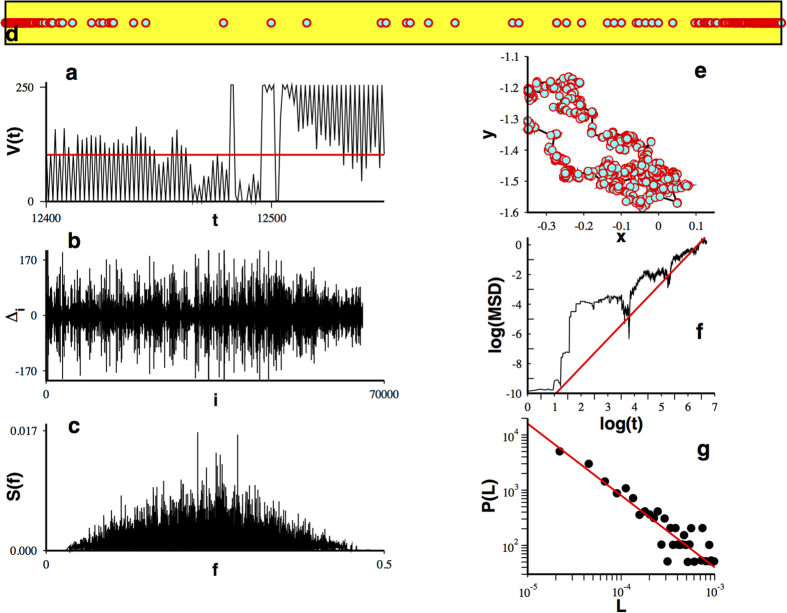
A superdiffusive Lévy random walk can be decoded from a real searching task. (**a**) From the temporal series of multichannel simultaneous recordings (here only a subset is shown) made from layer CA1 of the right dorsal hippocampus of three Long-Evans rats^45^,^46^, using the red threshold we can determine (**b**) a complex temporal behavior for the quantity 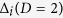 and its (**c**) power spectra calculated with 2^17^ points. (**d**) From the set of values satisfying 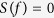 a fractal dust is determined and from the gaps in this fractal a (**e**) 2D random walk is induced. (**f**) Its mean squared displacement calculated as in Figure 2(g), evidencing superdiffusion; while its (**g**) steps distribution show it is a superdiffusive Lévy pattern (best fit in red).

Notwithstanding these outcomes it is still unclear if real experimentally determined neural time series would share the same dynamics as the biomathematical models analyzed above. We address this concern analyzing temporal series from multichannel simultaneous recordings made from layer CA1 of the right dorsal hippocampus of three Long-Evans rats during open field tasks in which the animals chased randomly placed drops of water or pieces of Froot Loops while on a elevated square platform^45^,^46^. Here we show results from one of these recordings. The temporal series (see Fig. 5a) was processed following the scheme II. Figure 5 resumes our findings: it can be seen that the analyzed experimental data behaves as expected: The Δ-series displays complex oscillations (Fig. 5b) and exhibits a power spectrum (see Fig. 5c, it has been calculated with a temporal series of 2^17^ points) whose zero power values allows us to extract a fractal set (Fig. 5d), with fractal dimension 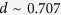, that induces a Levy walk on the plane (Fig. 5e) characterized by a Hurst exponent 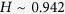 (Fig. 5f) and a power law distribution of steps with an exponent 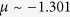 (Fig. 5g). Our approach seems to reveal an until now unknown coding mechanism based on a hidden multifractal. Indeed, it also points towards a relation between real neuronal dynamics and WLC, as evidenced by an excellent matching between theory and the outcomes obtained with the Long-Evans search related experimental temporal series. While these measures may not involve only motor related neurons we assume that the signal is carrying the relevant information related to the search process.

**Figure 6:**
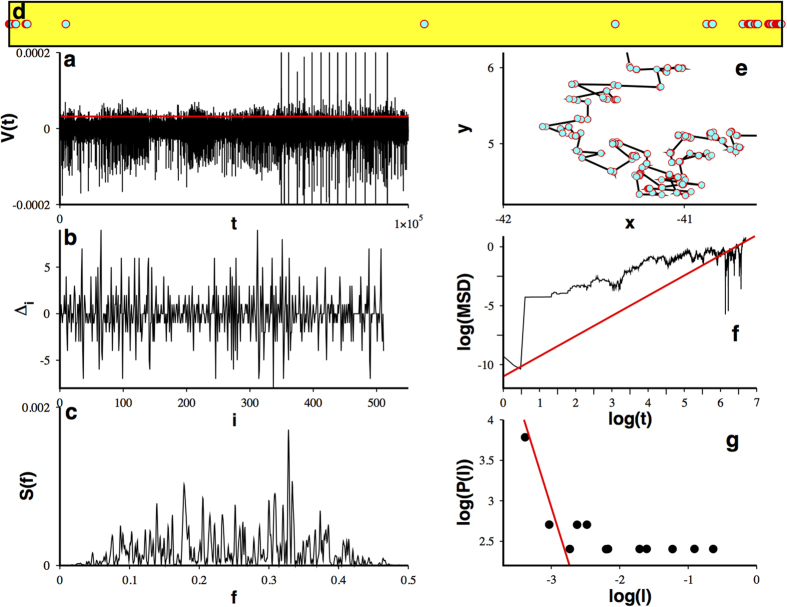
Superdiffusive random walks decoded from mice motor neurons. We extend our analysis to include the (**a**) action potential from adult mice (used threshold in red) whose (**b**) 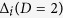 signal also behaves in a complex manner displaying a (**c**) power spectrum calculated with 2^10^ points with a diverse frequency content. From values of frequency satisfying 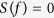 we can determine (**d**) a fractal dust whose gaps induces a (**e**) 2D random walk with (**f**) a superdiffusive mean square displacement calculated as in Figure 2(g). (**g**) The number of available data points used in the power spectra calculation is not enough to detect the possible Lévy character of this superdiffusion (best fit in red).

**Figure 7:**
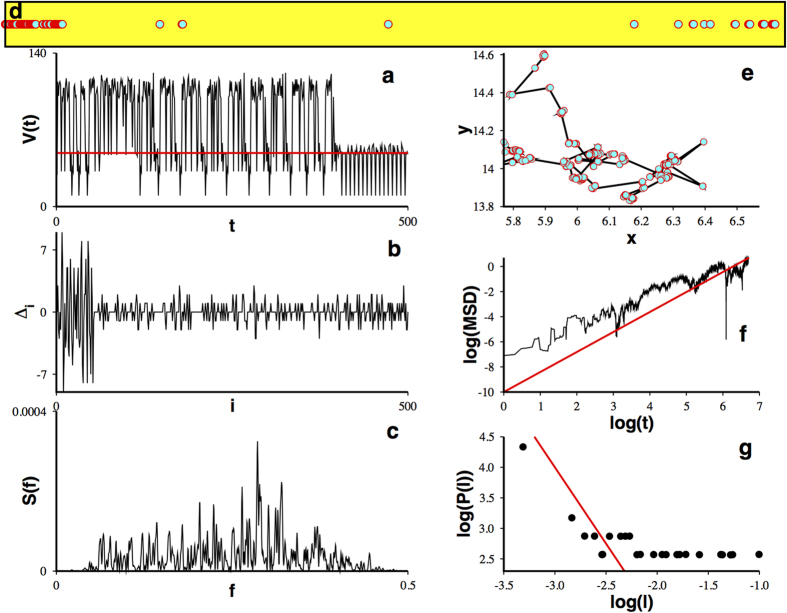
Superdiffusive random walks decoded from non-motor neurons: the case of grasshopper auditory neurons. A superdiffusive pattern can also be extracted from the (*Locusta migratoria*) auditory receptor (**a**) action potential (used threshold in red) whose (**b**) 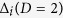 signal shows the characteristic complex fluctuations with a diverse (**c**) frequency content calculated with 2^10^ points determining (**d**) a fractal dust with dimension, 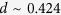 which gaps induce a (**e**) superdiffusive 2D random walk. (**f**) Mean squared displacement calculated as in Figure 2(g). (**g**) The number of available data points used in the power spectra calculation is not enough to detect the possible Lévy character of this superdiffusion (best fit in red).

To extend our analysis we decided to further explore different available datasets. Consequently, we analyzed extracellular recordings from the anterior motor cortex neurons related to voluntary movement in mice^47^ (see further details in the Supplementary Information). Results from this insight are summarized in Fig. 6. Again, a fractal set (this time with dimension 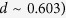 is obtained from the zero power values of the spectrum (calculated with a temporal series of 2^10^ points), inducing a superdiffusive random walk with a Hurst exponent 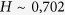. When analyzing the steps’ distribution it is found that the quality of the obtained profile is very poor. Even so, we attempt to fit a power law with exponent 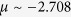. Obviously, this outcome is far from conclusive of a Levy superdiffusion random walk. While this latter case is not fully satisfactory, it can be said that the present approach has been successful unveiling the fractal code hidden in the two motor neuron’s series - i.e., both potentially related with searching - and obtaining (at least) superdiffusion. But, is this behavior exclusive of motor neurons or can it also be detected in non motor neurons - i.e., in neurons not directly related with searching - ? To answer this question, we analyze available data from experiments with the grasshopper (*Locusta migratoria*) auditory receptor cell^48^ (see Supplementary Information). Results for the grasshopper are shown in the Fig. 7 (the power spectrum was calculated with only 2^10^ available points). The behavior of the mean squared displacement correspond to a superdiffusive regime characterized by a Hurst exponent 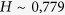 (Fig. 7f). However, the profile shown by the distribution of steps is insufficient to draw a solid conclusion. Again, we attempt to fit a power law, obtaining an exponent 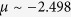. This result reveals that the superdiffusive behavior is not exclusive to motor neurons recordings and that they can also be obtained from auditory receptor cells.

## Discussion

It has been shown that WLC multifractality is an useful framework to generate neurologically based superdiffusive search patterns and to reproduce Lévy search patterns from a search related experimental temporal series (Long-Evans rats). Non conclusive results were obtained for Lévy search patterns in the cases of modeled and experimental neuronal time series not related with searching tasks. However, let’s note that this result could be caused by the limited number of points available to generate a Lévy random pattern. The power spectrum’s frequency precision is proportional to the number of data used in its calculation. In the cases of L-V and Long-Evans rats the temporal series were large enough to yield dense power spectra resulting in rich fractal dust. These sets were able to induce a random walk running on a diversity of steps lengths that were able to draw a well profiled power law. The detailed observation of the power spectra in Figs 4c,6c and 7c indicates that these power spectra do not show the frequency precision required to generate a fine grained fractal dust. Accordingly, one could expect to obtain Lévy search patterns also from larger temporal series in these situations. However, this can only be fully corroborated analyzing long temporal series. Thus, we have shown that Lévy search patterns can be obtained from non neurologically meaningful dynamics -the L-V equations- and real neuronal data related to a search process -the Long-Evans dataset-, while superdifussive random walks have been obtained in all the considered cases.

The conditions used in the Long-Evans (L-E) rat experiments do not seem to correspond to a search with revisitable targets as is required for an optimizing Lévy walk^23^. However, in this case once the rat drank the water the researchers dropped new drops randomly (György Buzsáki, private communication). While this fact does not exclude a particular spot to be filled again, chances are that this happened rarely. However, the animal has no information about this fact. Therefore, there is no reason to think that the animal is not revisiting small regions. It could happen if the water or food served in different instances fell randomly around the same area. Indeed, the rat would revisit such regions. Therefore we would not exclude revisiting dynamics in the L-E case. The biological mechanisms currently proposed to explain the generation of Lévy superdiffusion are not based on neuronal dynamics as the actual mechanism of WLC multifractality based coding does. The present approach may not show an evident relation with prioritization processes^49^ or with bursts of rapidly occurring reorientations^50^; two processes that have been related with the generation of superdiffusion^24^. In particular, in our case reorientation was dictated by an uniform distribution, i.e., unable to produce bursts of rapidly occurring reorientations. However, as it was already explained, in the current mechanism superdiffusion is generated by picking up randomly distances between the points of the fractal dust. This procedure may lead to a consecutive (bursting) extraction of large differences alternated with the consecutive (bursting) extraction of small ones. Therefore, one could conjecture the existence of some coincidences between the above mentioned ideas and the current mechanism.

In addition, a new approach to analyze coding in neurons has been presented. It may be possible to stimulate new experimental approaches to improve our insights into neuronal multifractal coding. The grasshopper results suggest that the underlying multifractality could be found at several processing levels, including receptor neurons. There is evidence that the metathoracic auditory system - to which auditory receptor neurons belong - could be a feedforward network^51^, however the presence of small but significant correlations between two receptor neurons does not allow to rule out the possibility of undetected weak synaptic contacts among receptors^51^, even though to present knowledge no synapses exist among them^52^. Our results favor the hypothesis that some sort of coupling between auditive receptors could be present. It seems important to recall pioneering results on the auditive receptor interaction of Locust that suggested electrotonic interaction among receptors in the region of the subreceptor plexus, given the absence of schwann sheaths and the penetration of receptor axons by collateral receptor axons^53^. Hence, it may be conjectured that auditive receptor neurons form a sensory network that support WLC multifractality. Notoriously, studies of sensory arrays mimicked by cellular automata excitable elements have shown collective nonlinear properties that improve input sensitivity and dynamic range^54^.

Instead of designing an experiment on the particular aspect of the generation of movement patterns perhaps it could be better to pay attention to the more general context of neuron coding. One may envisage that a possible experimental validation of this study could be achieved in a set up consisting of a neural system with a fractal dust precisely determined while the system controls a particular response. Subsequently, such a neural activity would be optogenetically turned off and the response triggered with a different simulated signal resembling the original one only in the fractal dust composition. An animal model for such a test could be the peripheral axons in the rat’s sciatic nerve where optical inhibition of motor neurons and muscle activity *in vivo*^55^,^56^ and in freely moving^57^ non-transgenic animals has been demonstrated. A possible limitation with this idea is the requirement of real-time monitoring of the local field of a population of neurons in the targeted branch of the rat’s sciatic nerve for its processing and fractal dust determination - this information would be used to produce the faked activation response after optical inhibition -. In this context it is still not clear how to achieve the process of faked signal injection or what would be the dimensionality of such a signal. No doubt the design of a proper experiment would need deeper insights to identify best fitted alternatives and/or possible simpler models.

Summarizing, evidence of a WLC multifractality based coding mechanism was presented. This phenomenon unveils a parameter-tuned fractal set that induces a 2D superdiffusive Lévy random walk for some of the cases analyzed. The current insight provides a theoretical ground to analyze modeled and real experimental neuronal time series. This work shows evidence about the possible neuronal basis of observed Lévy search patterns in animals. It should be remarked that the observed patterns are a result of tuning a control parameter. There are control parameter values for which a fractal dust able to induce superdiffusion can’t be obtained. We call attention to this point as it may be related to the fact that certain animals may not be following superdiffusive Lévy search patterns^18^,^24^ or displaying a multi-scale pattern^58^.

One could conjeture that if Lévy search patterns are an optimal searching solution, it could also be present in the computational exploration of a space of solutions associated to complex nervous system tasks. Obviously, a test of this conjeture requires further and deeper insights. Finally, it is well known that each spike carries information^59^. Remarkably, our approach is able to extract the information contained in the width of each spike -the duration of each bursting spike- to translate it into a detectable code. In this context, it must be noted that detectability is related to the determination of the constituent frequencies. However, from the point of view of nervous information processing, operating conditions based on long temporal series may be impractical. This suggest that the encoding mechanisms presented here may be well suited for better performance in a network context were each neuron generates its own (low-intermediate precision) fractal dust based on short temporal series, while the full network could induce well profiled Lévy walks superimposing all the generated single fractal dust. Our understanding of the discovered mechanism is still in an initial stage implying that it may contain a certain degree of speculation. Maybe the observed multifractality is also present in different nonlinear dynamical scenarios with the same coding potential.

## Methods

Simulation of the L-V map was done using the connectivity matrix specified in the Supplementary Information. The conductance model was numerically integrated with a backward differential formula (more details in the Supplementary Information). Peaks widths for the models and the experimental time series were determined fixing the threshold maximizing the number of widths. The Hurst exponents were determined using the Generalized Hurst exponent approach^60^ and the mean squared displacement was plotted based on the displayed random walk.

## Additional Information

**How to cite this article**: Gutiérrez, E. D. and Cabrera, J. L. A neural coding scheme reproducing foraging trajectories. *Sci. Rep*. **5**, 18009; doi: 10.1038/srep18009 (2015).

## Supplementary Material

Supplementary Information

## References

[b1] Shlesinger M. F. & Klafter J. Lévy walk versus Lévy flights In On Growth and Form (ed. Stanley H. E. & Ostrowsky N.) 279–283 (Martinus Nijhoff, 1986).

[b2] Viswanathan G. M., Afanasyev V., Buldyrev S. V. . Lévy flight search patterns of wandering albatrosses. Nature 381, 413–415 (1996). 10.1038/nature06199 17960243

[b3] Atkinson R. P. D., Rhodes C. J., Macdonald D. W. & Anderson R. M. Scale-free dynamics in the movement patterns of jackals. Oikos 98, 134–140 (2002).

[b4] Ma.rell A., Ball J. P. & Hofgaard A. Foraging and movement paths of female reindeer: Insights from fractal analysis, correlated random walks, and Lévy flights. Can. J. Zool. 80, 854–865 (2002).

[b5] Bartumeus F., Peters F., Pueyo S., Marrase C. & Catalan J. Helical Lévy walks: Adjusting searching statistics to resource availability in microzooplankton. Proc. Natl. Acad. Sci. USA 100, 12 771–12 775 (2003). 10.1073/pnas.2137243100 PMC240693 14566048

[b6] Ariel G. . Swarming bacteria migrate by Lévy Walk. Nat. Commun. 6, 9396 (2015). 10.1038/ncomms9396 PMC4598630 26403719

[b7] Ramos-Fernández G., Mateos J. L., Miramontes O., Germinal C., Larralde H. & Ayala-Orozco B. Lévy walk patterns in the foraging movements of spider monkeys (*Ateles geoffroyi*). Behav. Ecol. Sociobiol. 55, 223–230 (2004).

[b8] Boyer D., Miramontes O., Ramos-Fernández G., Mateos J. L. & Cocho G. Modeling the searching behavior of social monkeys. Physica A 342, 329–335 (2004).

[b9] Reynolds A. M. Optimal scale-free searching strategies for the location of moving targets: New insights on visually cued mate location behaviour in insects. Phys. Lett. A 360, 224–227 (2006).

[b10] Johnson S. N. . Non-invasive techniques for investigating and modelling root-feeding insects in managed and natural systems. Agric. and Forest Entomol. 9, 39–46 (2007).

[b11] Reynolds A. M., Smith A. D., Menzel R., Greggers U., Reynolds D. R. & Riley J. R. Displaced honey bees perform optimal scale-free search flights. Ecology 88, 1955–1961 (2007). 17824426 10.1890/06-1916.1

[b12] DeKnegt H. J., Hengeveld G. M., VanLangevelde F., deBoer W. F. & Kirkman K. P. Patch density determines movement patterns and foraging efficiency of large herbivores. Behav. Ecol. 18, 1065–1072 (2007).

[b13] Zhang X., Johnson S. N., Crawford J. W., Gregory P. J. & Young I. M. A general random walk model for the leptokurtic distribution of organism movement: Theory and application. Ecol. Model. 200, 79–88 (2007).

[b14] Seuront L., Duponchel A.-C. & Chapperon C. Heavy-tailed distributions in the intermittent motion behaviour of the intertidal gastropod *Littorina littorea*. Physica A 385, 573–582 (2007).

[b15] Reynolds A. M. & Frye M. A. Free-flight odor tracking in Drosophila is consistent with an optimal intermittent scale-free search. PLoS ONE 2, e354 (2007). 17406678 10.1371/journal.pone.0000354 PMC1831497

[b16] Sims D. W., Witt M. J., Richardson A. J., Southall E. J. & Metcalfe J. D. Encounter success of free-ranging marine predator movements across a dynamic prey landscape. Philos. Trans. R Soc. London Biol. 273, 1195–1201 (2006). 10.1098/rspb.2005.3444 PMC1560279 16720391

[b17] Sims D. W. . Scaling laws of marine predator search behaviour. Nature 451, 1098–1102 (2008). 18305542 10.1038/nature06518

[b18] Humphries N. E. . Environmental context explains Lévy and Brownian movement patterns of marine predators. Nature 465, 1066–1069 (2010). 20531470 10.1038/nature09116

[b19] Focardi S., Montanaro P. & Pecchioli E. Adaptative Lévy walks in foraging fallow deer. PLoS ONE 4, e6587 (2009). 19668369 10.1371/journal.pone.0006587 PMC2719089

[b20] Raichlen D. A., Wood B. M., Gordon A. D., Mabulla A. Z. P., Marlowe F. W. & Pontzer H. Evidence of Lévy walk foraging patterns in human hunter-gatherers. Proc. of the Nat. Acad. Sci. USA 111, 728–733 (2014). 10.1073/pnas.1318616111 PMC3896191 24367098

[b21] Stephens D. W. & Krebs J. R. Foraging Theory (Princeton University Press, Princeton, 1986).

[b22] Garcia R. . Optimal foraging by zooplankton within patches: The case of Daphnia. Math, Biosci. 207, 165–188 (2007). 17363010 10.1016/j.mbs.2006.11.014

[b23] Viswanathan G. M. . Optimizing the success of random searches. Nature 401, 911–914 (1999). 10553906 10.1038/44831

[b24] Viswanathan G. M., da Luz M. G. E. , Raposo E. P. & Stanley H. E. The physics of foraging: an introduction to random searches and biological encounters. (Cambridge University Press, Cambridge, 2011).

[b25] Mirny L. . How a protein searches for its site on DNA: The mechanism of facilitated diffusion. J. Phys. A, 42, 434013 (2009).

[b26] Cressoni J. C., da Silva M. A. A. & Viswanathan G. M. Amnestically induced persistence in random walks. Phys. Rev. Lett. 98, 070603 (2007). 17359007 10.1103/PhysRevLett.98.070603

[b27] Bartumeus F. & Levin S. A. Fractal reorientation clocks: Linking animal behavior to statistical patterns of search. Proc. Natl. Acad. Sci. USA 105, 19072–19077 (2008). 19060198 10.1073/pnas.0801926105 PMC2614717

[b28] Martin J. R., Faure P. & Ernst R. The power law distribution for walking-time intervals correlates with the ellipsoid-body in Drosophila. J. Neurogenet. 15, 205–219 (2001). 12092904 10.3109/01677060109167377

[b29] May R. M. & Leonard W. Nonlinear aspects of competition between three species. SIAM J. Appl. Math. 29, 243–253 (1975).

[b30] Rabinovich M. I., Volkovskii A., Lecanda P., Huerta R., Abarbanel H. D. I. & Laurent G. Dynamical Encoding by Networks of Competing Neuron Groups: Winnerless Competition. Phys. Rev. Lett. 87, 068102 (2001). 11497865 10.1103/PhysRevLett.87.068102

[b31] Rabinovich M. I., Afraimovich V. S., Bick C. & Varona P. Information flow dynamics in the brain. Phys. Life Rev. 9, 51–73 (2012). 22119154 10.1016/j.plrev.2011.11.002

[b32] Laurent G. Olfactory network dynamics and the coding of multidimensional signals, Nature Rev. Neurosci. 3, 884–895 (2002). 12415296 10.1038/nrn964

[b33] Ashwin P. & Timme M. Nonlinear dynamics: When instability makes sense. Nature 436, 36 (2005). 16001052 10.1038/436036b

[b34] Baeg E. H., Kim Y. B., Huh K., Mook-Jung I., Kim H. T. & Jung M. W. Dynamics of population code for working memory in the prefrontal cortex. Neuron 40, 177–188 (2003). 14527442 10.1016/s0896-6273(03)00597-x

[b35] Uchida N. & Mainen Z. F. Speed and accuracy of olfactory discrimination in the rat. Nature Neurosci. 6, 1224–1229 (2003). 14566341 10.1038/nn1142

[b36] Mazor O. & Laurent G. Transient dynamics versus fixed points in odor representations by locust antennal lobe projection neurons. Neuron 48, 661–673 (2005). 16301181 10.1016/j.neuron.2005.09.032

[b37] Jones L. M., Fontanini A., Sadacca B. F., Miller P. & Katz D. B. Natural stimuli evoke dynamic sequences of states in sensory cortical ensembles. Proc. Natl. Acad. Sci. USA 104, 18772–18777 (2007). 18000059 10.1073/pnas.0705546104 PMC2141852

[b38] Rabinovich M. I., Huerta R., Varona P. & Afraimovich V. S. Transient cognitive dynamics, metastability, and decision making. PLoS Comput. Biol. 4, e1000072 (2008). 18452000 10.1371/journal.pcbi.1000072 PMC2358972

[b39] Buzsáki G. Neural syntax: cell assemblies, synapsembles, and readers. Neuron 68 362–385 (2010). 21040841 10.1016/j.neuron.2010.09.023 PMC3005627

[b40] Varona P., Levi R., Arshavsky Y. I., Rabinovich M. I. & Selverston A. I. Competing sensory neurons and motor rhythm coordination. Neurocomputing 58, 549–554 (2004).

[b41] González-Díaz L., Gutiérrez E. D. & Cabrera J. L. Winnerless competition in coupled Lotka-Volterra maps. Phys. Rev. E. 88, 012709 (2013). 10.1103/PhysRevE.88.012709 23944494

[b42] Chlabra A. B., Meneveau C., Jensen R. V. & Sreenivasan K. R. Direct determination of the *f*(*α*) singularity spectrum and its application to fully developed turbulence. Phys. Rev. A 40, 5284–5294 (1989). 9902794 10.1103/physreva.40.5284

[b43] Vicsek T. Fractal Growth Phenomena. (World Scientic, Singapore, 1989).

[b44] Tristan I., Rulkov N. F., Huerta R. & Rabinovich M. I. Timing control by redundant inhibitory neuronal circuits. Chaos 24, 013124 (2014). 24697386 10.1063/1.4866580 PMC3977790

[b45] Mizuseki K., Sirota A., Pastalkova E. & Buzsáki G. Theta oscillations provide temporal windows for local circuit computation in the entorhinal-hippocampal loop. Neuron 64, 267–280 (2009). 19874793 10.1016/j.neuron.2009.08.037 PMC2771122

[b46] Mizuseki K., Sirota A., Pastalkova E. & Buzsáki G. (2009): Multi-unit recordings from the rat hippocampus made during open field foraging. http://dx.doi.org/10.6080/K0Z60KZ9.

[b47] Li Z. V. . Flow of cortical activity underlying a tactile decision in mice. Neuron 81, 179–194 (2014). 24361077 10.1016/j.neuron.2013.10.020 PMC3984938

[b48] Rokem . Spike-Timing Precision Underlies the Coding Efficiency of Auditory Receptor Neurons. Journal of Neurophysiology 95, 2541–2552 (2006) 16354733 10.1152/jn.00891.2005

[b49] Vazquez A., Oliveira J. G., Dezso Z., Goh K.-I., Kondor I. & Barabási A.-L. Modeling bursts and heavy tails in human dynamics. Phys. Rev. E 73, 036127 (2006). 10.1103/PhysRevE.73.036127 16605618

[b50] Bartumeus F., da Luz M. G. E., Viswanathan G. M. & Catalan J. Animal search strategies: A quantitative random-walk analysis. Ecology 86, 3078–3087 (2005).

[b51] Vogel A. & Ronacher B. Neural correlations increase between consecutive processing levels in the auditory system of locust. J. Neurophysiol. 97, 2280–2288 (2015). 10.1152/jn.00796.2006 17360818

[b52] Rehbein H. Auditory neurons in the ventral cord of the locust: morphological and functional properties. J. Comp. Physiol. 110, 233–250 (1976).

[b53] Popov A. V. & Svetlogorskaya I. D. Receptor interaction and ultrastructural organization of the auditory nerve inLocusta migratoria. Neurosci. Behav. Physiol. 5, 0097–0549 (1972). 10.1007/BF01184808 4656001

[b54] Copelli M., Roque A. C., Oliveira R. F. & Kinouchi O. Physics of psychophysics: Stevens and Weber-Fechner laws are transfer functions of excitable media. Phys. Rev. E 65, 060901 (2002). 10.1103/PhysRevE.65.060901 12188696

[b55] Liske H. . Optical inhibition of motor nerve and muscle activity *in vivo*. Muscle & Nerve 47, 916–921 (2013). 23629741 10.1002/mus.23696 PMC5507203

[b56] Liske H., Qian X., Anikeeva P., Deisseroth K. & Delp S. Optical control of neuronal excitation and inhibition using a single opsin protein ChR2. Sci. Rep. 3, 03110 (2013). 10.1038/srep03110 PMC3813941 24173561

[b57] Towne C., Montgomery K. L., Iyer S. M., Deisseroth K. & Delp S. L. Optogenetic Control of Targeted Peripheral Axons in Freely Moving Animals. PLoS ONE 8 e72691. (2013). 23991144 10.1371/journal.pone.0072691 PMC3749160

[b58] Benhamou S. Of scales and stationarity in animal movements. Ecol. Lett. 17, 261–272 (2014). 24350897 10.1111/ele.12225

[b59] Borst A. & Theunissen F. E. Information theory and neural coding. Nat. Neuro. 2, 947–957 (1999). 10.1038/14731 10526332

[b60] Di Matteo T. Multi-scaling in finance. Quant. Financ. 7, 21–36 (2007).

