# Supplementary material for: A neural coding scheme reproducing foraging trajectories
Source: Sci Rep. 2015 Dec 9;5:18009. doi: 10.1038/srep18009 (PMC4673616; doi:10.1038/srep18009)
Supplement: Supplementary Information [file srep18009-s1.pdf]

Supplementary material of

"A neural coding scheme reproducing foraging trajectories"

Esther D. Gutiérrez & J. L. Cabrera

Laboratorio de Dinámica Estocástica, Centro de Física, Instituto Venezolano de Investigaciones Científicas. Caracas 1020-A, Venezuela

I. L-V MODEL

Simulations of the L-V map model with $N = 6$ were done using the following connectivity matrix:

$$\rho_{ij} = \begin{pmatrix} 1 & 1.05 & 1.001 & 1.001 & 1.001 & 0 \\ 0 & 1 & 1.15 & 1.001 & 1.001 & 1.001 \\ 1.001 & 0 & 1 & 1.14 & 1.001 & 1.001 \\ 1.001 & 1.001 & 0 & 1 & 1.07 & 1.001 \\ 1.001 & 1.001 & 1.001 & 0 & 1 & 1.09 \\ 1.001 & 1.001 & 1.001 & 1.001 & 0 & 1 \end{pmatrix},$$

Where, the initial conditions are $a_i(t = 0) = (0.0001, 0.0001, 0.0001, 0.0001, 0.04, 0.0001)$.

II. CONDUCTANCE BASED NEURONAL MODEL

We used the conductance based neuronal model showing WLC given by [1] based in the model of Carter et al. [2] as modified by R. Huerta (more details in http://senselab.med.yale.edu/modeldb/ShowModel.asp?model=145162). Used parameters values were: $N = 3$, $V_t = -57$mV, $I_{dc} = 0$ and $g_{GABA_B} = 10$ns. Peaks width were determined with a threshold value of $\tau = \frac{1}{2}V_{max} = \frac{1}{2}.14.3484 = 7.1742$ mV. Integration of the 36 stiff ODE was achived with a backward differential formula with Newton iteration from the library CVODE (http://computation.llnl.gov/casc/sundials/description/description.html)

III. LONG-EVANS RATS DATA

The data set contains multichannel simultaneous recordings made from layer CA1 of the right dorsal hippocampus of three Long-Evans rats during open field tasks in which the

animals chased randomly placed drops of water or pieces of Froot Loops while on a elevated square platform [3, 4] . For our study we used the smallest size session data (directory ec013.527). Datafiles were obtained from the *Collaborative Research in Computational Neuroscience* Data sharing repository (crcns.org). Peaks width were determined with a threshold value of $\tau = 0.4V_{max} = 0.4$ x $255 = 102$ x spike amplitude.

IV. MICE MOTOR NEURON DATA

Temporal series from experiments with motor neuron data were obtained from the *Collaborative Research in Computational Neuroscience* Data sharing repository (crcns.org) . In particular we used data from extracellular recordings from anterior lateral motor cortex (ALM) neurons of adult mice performing a tactile decision behavior [5, 6]. Data was contributed by Nuo Li in the lab of Karel Svoboda at Janelia Farm. Peaks width were determined with a threshold value of $\tau = 1.0$ x 106 mV.

V. LOCUSTA MIGRATORIA DATA

This data set contains recordings from the grasshopper (*Locusta migratoria*) auditory receptor cell [7–10] obtained from the *Collaborative Research in Computational Neuroscience* Data sharing repository (crcns.org) . All sets of available data were processed and results qualitatively similar to those reported in the paper were obtained . The paper figure was done with data from the first dataset. Peaks width was determined with a threshold value of $\tau = 0.4V_{max} = 0.4$ x 122 mV = 48.8 mV.

---

[1] Tristan, I., Rulkov, N. F., Huerta, R. & Rabinovich, M. I. Timing control by redundant inhibitory neuronal circuits. *Chaos* **24**, 013124 (2014).

[2] Carter M.E. et al Mechanism for Hypocretin-mediated sleep-to-wake transitions *Proc. Nat. Acad. of Sci.* **109**, E2635E2644 (2012).

[3] Mizuseki K., Sirota A., Pastalkova E., Buzsáki G., *Neuron* **64**, 267-280 (2009).

[4] Mizuseki K, Sirota A, Pastalkova E, Buzsáki G. *Multi-unit recordings from the rat hippocampus made during open field foraging.* (2009) Available at: CRCNS.org. http://dx.doi.org/10.6080/K0Z60KZ9 .

[5] Li, Z.V., *et al.* Flow of cortical activity underlying a tactile decision in mice. *Neuron* **81**, 179 - 194 (2014).

[6] Li, N., Gerfen, C. R., Svoboda K., *Extracellular recordings from anterior lateral motor cortex (ALM) neurons of adult mice performing a tactile decision behavior.* (2014) Available at: CRCNS.org. http://dx.doi.org/10.6080/K0RF5RZT .

[7] Rokem, A., Watzl, S., Gollisch, T., Stemmler, M., Herz, A. V. M. and Samengo, I. *Recordings from grasshopper (Locusta Migratoria) auditory receptor cells.* (2009) Available at: CRCNS.org. http://dx.doi.org/10.6080/K0BG2KWB.

[8] Rokem, et al. Spike-Timing Precision Underlies the Coding Efficiency of Auditory Receptor Neurons. *J. Neurophys.* **95**, 2541-2552 (2006).

[9] Eyherabide, H.G., Rokem, A. , Herz, A.V.M. and Samengo, I. Burst Firing as a Neural Code in an Insect Auditory System. *Front. Hum. Neurosci.*, **3** 1 (2008).

[10] Eyherabide, H.G., Rokem, A. , Herz, A.V.M. and Samengo, I. Bursts generate a non-reducible spike-pattern code. *Front. Neurosci.*, **3** 8-14 (2009).
